# Strengthening the Aging Workforce: Building Sustainable Partnerships for Innovative, Skill-Based Training

**DOI:** 10.1093/geroni/igaf122.1039

**Published:** 2025-12-31

**Authors:** Bronwyn Keefe

**Affiliations:** Boston University School of Social Work, Boston, Massachusetts, United States

## Abstract

This presentation will describe workforce development partnerships between an aging Center within a large private University and state and community-based agencies that provide health and social services to older adults. The aging workforce is looking to build their competencies just as state and local agencies seek partnerships with academic institutions to offer standardized, high-quality, skill-based learning for their employees. Many practitioners in the field of aging have not received formal education in working with older adults, while those who seek services are more diverse than ever and have a complexity of medical and social concerns. Reciprocal academic and community-based partnerships are essential to prepare and retool the aging workforce, especially during a great exodus of social service practitioners. This presentation will highlight the impact of workforce development initiatives through offering innovative and evidence-informed programs. We will share state-specific examples that have led to state policy and funding to strengthen the aging workforce, which has sustained the Center’s efforts for over 10 years. Training outcomes are measured through multiple mechanisms, e.g., pre-post competency assessments, impact on knowledge and skills, practice change, and overall satisfaction with the program. Results show statistically significant gains in key competency areas, including conducting assessments, identifying resources, providing culturally appropriate and person-centered care, and supporting clients during care transitions. This presentation will highlight the importance of statewide workforce development programs in providing essential skills needed to deliver high-quality care and services to older adults and how this has led to building a self-sustaining Center.

